# Exploiting Interfacial Effects between Collapsing Bubbles and Nanocarbon/TiN Substrates for the Green Synthesis of Self-Organized Noble Metal and Nanoalloy Nanoparticles

**DOI:** 10.3390/mi14061141

**Published:** 2023-05-28

**Authors:** Mohammed Es-Souni

**Affiliations:** Institute for Materials & Surface Technology (IMST), 24149 Kiel, Germany; mohammed.es-souni@fh-kiel.de

**Keywords:** noble metal nanoparticle, multi-material, surface modification, catalyst, electrocatalysis

## Abstract

Noble metal nanoparticles and multi-materials thereof are processed on a substrate from aqueous solutions of the metallic ions, precluding any chemical additives/catalysts. The methods reported here take advantage of interactions between collapsing bubbles and the substrate that result in the generation of reducing radicals at the substrate surface and leading to the reduction of the metal ions on those sites, followed by nucleation and growth. Two selected substrates where these phenomena take place are nanocarbon and TiN. By either using ultrasonic radiation of the substrate in ionic solution or quenching the substrate in a solution from temperatures above the Leidenfrost temperature, a high density of nanoparticles of Au, Au/Pt, Au/Pd and Au/Pd/Pt are synthesized on the substrate surface. The sites where the reducing radicals are generated determine the self-assembly of the nanoparticles. The methods yield highly adherent surface films and nanoparticles; they are materials efficient and cost effective because only the surface is modified with costly materials. The formation mechanisms of these green multi-material NPs are described. Outstanding electrocatalytic performances in acidic solutions of methanol and formic acid are demonstrated.

## 1. Introduction

Noble metal nanoparticles (NMNPs) are crucial to various cutting-edge technologies, including biotechnology, sensing, and catalysis [1,2,3,4,5]. In most of the work published so far, NPs are processed in the solution from common precursors using specific reducing chemicals; surfactants and capping agents may be advantageously added to promote preferential growth of crystal facets [6]. The NPs may be immobilized on a substrate surface to take advantage of their coupled optical properties, e.g., for surface-enhanced Raman (SERS) or infrared spectroscopy, or for their collective behaviour in catalytic applications [7,8,9,10,11]. While sophisticated top-down methods, such as electron beam lithography, are occasionally used to process highly conformal nanostructures on a substrate with well-defined dimensions, morphology, and gap [7,12,13], bottom-up techniques have been the focus in most research. The NPs are usually synthesized and functionalized in solution and self-assembled on substrates with specific functional groups. These methods have demonstrated their versatility and capability to self-assemble ordered nanostructures of a variety of materials on a substrate [13,14,15]. Another bottom-up technique that is of particular interest relies on solid templates, especially porous anodized alumina (PAA) templates, for making large-area ordered nanomaterials with different aspect ratios and, to some extent, morphology [16,17]. Mostly, the nanomaterials are deposited into the pores via electrochemical deposition from suitable electrolytes. The technique is straightforward because the porous template molds the deposited material into the predefined pore dimension and periodicity. Moreover, it is cost-effective because of low investment and consumable costs. Of particular interest are thin film aluminum oxide templates that are processed on a substrate (glass, silicon, etc.), which allow the fabrication of on-substrate, self-standing ordered nanostructures. For instance, these thin film templates were used to fabricate Au- and Ag Nanorods (NRs) for SERS applications [8,18], catalytic nanostructures based on porous Pt-nanorods [10], as well as NiO-nanotubes for energy storage [19]. More applications involved scratch resistant, transparent anti-biofouling films [20,21].

In the present paper, two powerful, high-yield, and environmentally sustainable processing methods of on-substrate NMNPs and nanoalloys are presented. The NPs are directly synthesized on two selected substrates that are frequently used for catalytic and energy storage applications. Processing proceeds directly from aqueous solutions without the need of reducing chemicals. Surfactants may be added but are not necessary. Both methods rely on the interactions at the interface between collapsing cavities and the substrate surface that lead to the generation of highly reducing radicals. Reduction of metallic ions and nucleation of metallic clusters occur on the sites of collapsing cavities which also determine the sites of NP self-assembly. For catalytic applications, the usual procedure is to process NPs in solution, and then load a certain amount of them onto a support-electrode material, mostly carbon based, e.g., Vulcan, glassy carbon, etc.; this entails the risk of damaging the particles and agglomeration. The current literature abounds with processing protocols of NMNPs and their multi-materials, discussed using the procedure described above. Few examples with relevance to the present work may be found in the references [22,23,24,25]. Whether the methods are based on microemulsion and reduction [24] or multistep reduction [23,25], they all require lots of chemicals and multiple steps before the nanomaterials can be filtrated, assembled on a catalyst support, and tested. In contrast, the processes described in this paper forgo these steps because the particles are directly reduced on the support. Further, they require fewer chemicals, lesser amounts of expensive precious metals, and are in this respect materials efficient. It will be demonstrated that large surfaces of supported porous nanocarbon and TiN films can be modified with noble metals (Au, Pt and Pd), NPs, and multi-materials NPs that are, among others, critical for biomedical [3,26] and catalysis applications. It will be further shown that any material that could be surface modified with a layer of carbon nanotubes, or another carbon nanomaterial can be used as support for the processing of NMNPs.

## 2. Experimental Section

### 2.1. Supported Porous Nanocarbon and TiN Film Fabrication

For nanocarbon film fabrication, mill-finish stainless steel substrates were dip coated in a precursor solution consisting of 15 wt. % Polyvinylidene fluoride (PVDF), 12 wt. % Ammonium nitrate (NH_4_NO_3_) and 1 wt. % Multi-walled carbon nanotubes (MWCNT) dissolved in N,N-Dimethylformamide (C_3_H_7_NO). By tempering at 150 °C, porous PVDF-MWCNT-nanocomposite films were obtained. Pyrolysis of these films at 550 °C under nitrogen atmosphere yielded porous nanocarbon-MWCNT-nanocomposite films. A detailed process description can be found in our earlier work [27,28].

The TiN films of approximately 500 nm thickness were deposited on stainless steel substrates using magnetron sputtering from a TiN-target (Goodfellow, Cambridge, UK) in a physical vapor deposition device (PVD75, Lesker, Jefferson Hills, PA, USA). The substrate temperature was 250 °C and the deposition rate 0.07 Å/s at a power of 220 W.

### 2.2. Surface Modification of Supported Porous Nanocarbon and TiN Films

(a) Modification via sonochemistry [29]: Sonication experiments were performed using a Bandelin Sonorex RK31 (Schalltec, Welersteusslingen, Germany) bath with an ultrasonic nominal output of 40 W and ultrasonic frequency of 35 kHz. For the processing of Au-NPs, the substrate was immersed either in a 2- or 1-mM solution of HAuCl_4_ and sonicated for 90 s. For the AuPd and PtPd NPs, a 1:1 mixture of HAuCl_4_ and K_2_PdCl_4_ (H_2_PtCl_6_) was used under the same conditions.

(b) Modification via dipping in a pre-heated substrate above the Leidenfrost-temperature.

The porous nanocarbon-MWCNT-nanocomposite films were heated to 300 ± 10 °C and directly quenched in an aqueous solution of polyethylene-glycol functionalized single walled carbon nanotubes (SWCNTs, Sigma-Aldrich, Taufkirchen, Germany) to achieve a SWCNT-film on the surface. Surface modification with noble metal NPs followed a similar procedure, where the SWCNT modified substrates were heated again and quenched in the aqueous solutions of the metallic ions. An aqueous solution of 2 mM of HAuCl_4_ was used for Au-NPs, 2 mM H_2_PtCl_6_ for Pt-NPs, and 2 mM K_2_PdCl_4_ for Pd-NPs. Sequential heating and quenching in the solutions of different materials yielded multi-material modified surfaces (see below). Samples were also prepared for the scanning transmission electron microscopy (STEM) investigations. For this purpose, Ni-TEM grids were fixed on a stainless steel substrate using stainless steel screws, and the same procedure as above was applied to them, i.e., modification with SWCNT from a colloidal solution, followed by sequential modification with noble metal NPs.

### 2.3. Characterization

The structure was analysed by X-ray diffraction (X’Pert Pro diffractometer PANalytical, Eindhoven, Holland) in grazing incidence diffraction mode with constant θ = 1° using monochromatic Cu Kα radiation with λ = 1.5418 Å and a scanning range between 10° and 90° (2θ). The microstructure and morphology were characterized with a high-resolution scanning electron microscope (SEM, Ultra Plus, ZEISS, Oberkochen, Germany) operating in the secondary (SE) and energy-selective backscattered electron (ESB) modes. The SEM was also equipped with a scanning transmission electron microscopy (STEM) detector. High-resolution SE and ESB images were obtained with 2 kV acceleration voltage whereas the STEM investigations were conducted with 25 kV.

To further bolster the result that Au-NPs form exclusively in the presence of the substrate, UV–vis spectra were recorded versus H_2_O using a spectrophotometer (Lambda 35, Perkin-Elmer, Waltham, MA, USA). Sonication was conducted in a 1 mM HAuCl_4_ solution, separately in the presence of the substrate and in its absence.

The electrocatalytic experiments were conducted using an electrochemical workstation (ZAHNER IM6e, Kronach, Germany) in a three-electrode setup with a Pt mesh and HydroFlex (reversible H_2_ reference electrode) as counter and reference electrodes, respectively. First, cyclic voltammetry (CV) measurements were performed in 0.5 M H_2_SO_4_. For formic acid and methanol electrooxidation 0.5 M aqueous solutions in 0.5 M H_2_SO_4_ were used. The potentials are referred to as the normal hydrogen electrode (NHE).

All electrolytes in the electrochemical experiments were saturated with bubbling nitrogen for at least 30 min before use.

## 3. Results

For each processing method, the results are presented first, followed by a discussion of the formation mechanisms of the different nanostructures. It will be shown that the operating mechanisms are rather similar.

### Structure and Morphology

(a) Supported Au and nanoalloy NPs by sonochemistry.

The supported porous carbon-MWCNT-nanocomposite films are obtained following the procedure described in the experimental section. The films are approximately 2 µm thick and contain a wide range of pore sizes, from macro- to mesopores [28]. They are hydrophobic with a reproducible water contact angle (WCA) of approximately 140° (see below). In contrast, the TiN-films are rather hydrophilic with a WCA of approximately 40°.

**Figure 1:**
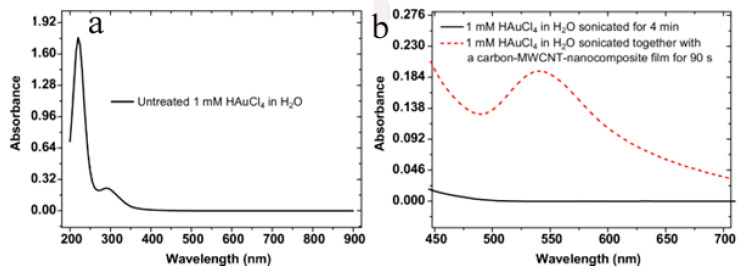
(**a**) absorption spectrum of a 1 mM aqueous solution of HAuCl_4_ (pH: 2.91) showing the absorption bands of AuCl_4_^−^ at 223 and 307 nm; (**b**) compares the absorption spectrum of the sonicated solution in the presence and absence of the substrate. The dotted line curve shows the plasmonic absorption of Au-NPs centred around 540 nm that is obtained when the solution is sonicated in the presence of the substrate.

**Figure 2:**
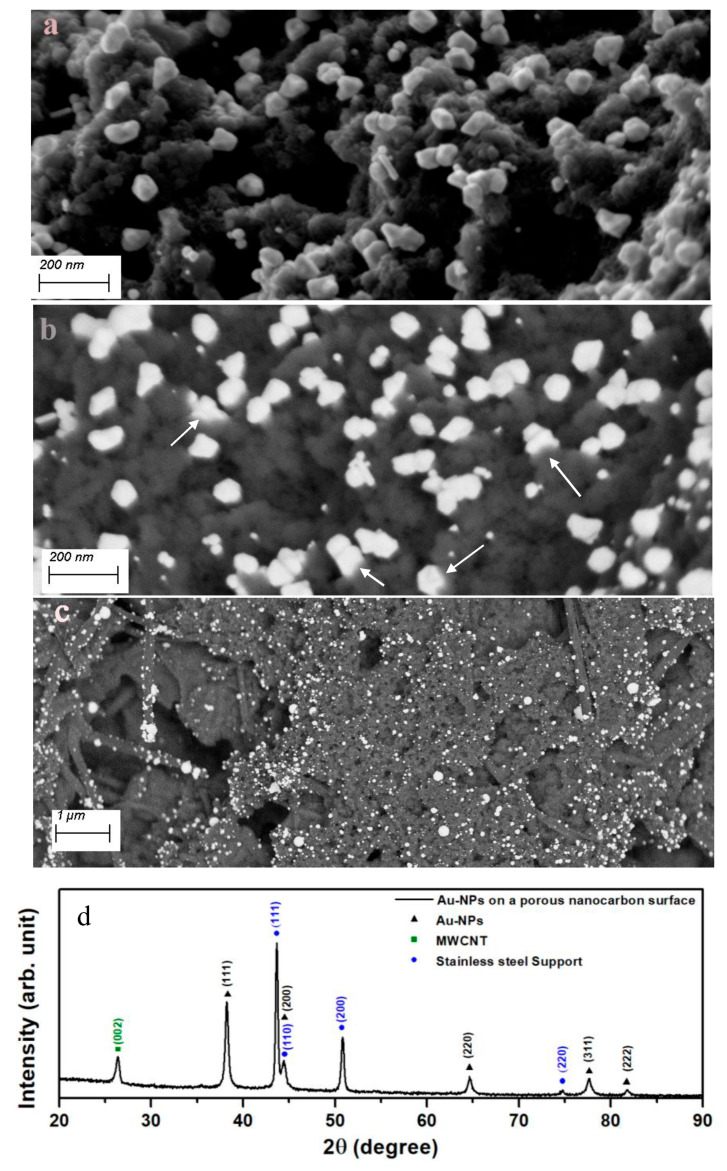
Secondary electron (SE) micrograph of the Au-NPs obtained after 90 s sonication of the porous nanocarbon-MWCNTs film in a 2 mM aqueous solution of HAuCl_4_ (**a**); (**b**) shows an ESB micrograph in which the bonding between the Au-NPs and the substrate surface may be suggested (arrows); (**c**) is an ESB micrograph of Au-NPs after 90 s sonication, followed by a 30 s run; (**d**) grazing incidence XRD pattern showing the main reflexes of Au, the substrate (mainly the graphitic MWCNTs), and the stainless steel support.

**Figure 3:**
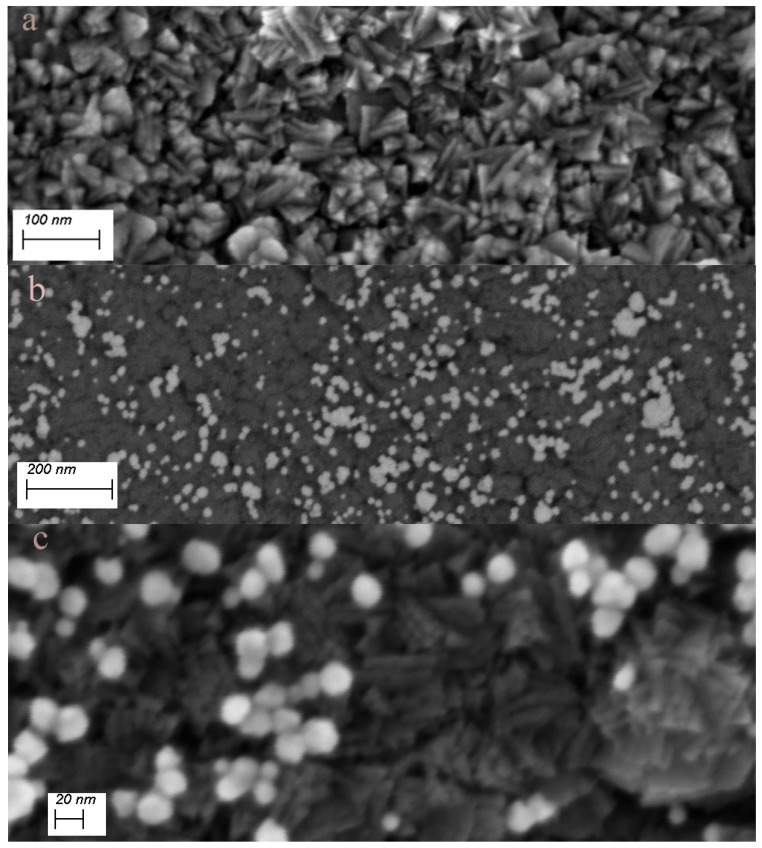
(**a**) SE micrograph of the TiN substrate surface showing the morphology of the TiN-crystallites; (**b**) ESB micrograph of Au-NPs on TiN after 90 s sonication in 2 mM solution of HAuCl_4_; (**c**) higher-resolution SE micrograph showing the Au-NPs and the underlying TiN-substrate.

Previous reports had shown that sonication of an aqueous solution containing HAuCl_4_ did not yield Au-NPs in the absence of catalysts [30,31]. The author’s own observations show the same results using a 2 mM HAuCl_4_ solution sonicated for as long as 300 s in the absence of the nanocarbon or TiN substrates. This is readily inferred from the photometry spectrum of the solution, which shows only the transitions of the AuCl_4_^−^ complex [32], shown in Figure 1a. A completely different outcome is obtained in the presence of a porous nanocarbon substrate; Au-NPs had already formed on the surface after 60 s sonication. As Figure 2 illustrates, a range of size distribution of the NPs from 10 to 100 nm and various morphologies are obtained. The ESB micrograph, Figure 2b, also suggests that the particles tightly stick to the substrate, and indeed, a renewed sonication in the HAuCl_4_ solution would only add to the density of the particles and their growth, Figure 2c. Moreover, Au-NPs also form in the solution, as illustrated in Figure 1b, where the absorption peak centered at 540 nm, which is characteristic for Au-NPs, is clearly seen. Similar results were obtained with the TiN-terminated substrates, shown in Figure 3.

A previous publication [29] had shown that nanoalloys of PtPd and AuPd were easily processed on both substrates via sonication in a mixture of aqueous solutions of either H_2_PtCl_6_ and K_2_PdCl_4_ or HauCl_4_ and K_2_PdCl_4_. The alloy composition may be controlled by the mixing ratio of the respective solutions. In [29], nanoalloys in close range to PtPd27 and AuPd22 were obtained by mixing equal volumes of 2 mM solutions of H_2_PtCl_6_ and K_2_PdCl_4_ or HAuCl_4_ and K_2_PdCl_4_.

(b) supported NMNPs and nanoalloys via a Leidenfrost-mediated reduction of metal ions

**Figure 4:**
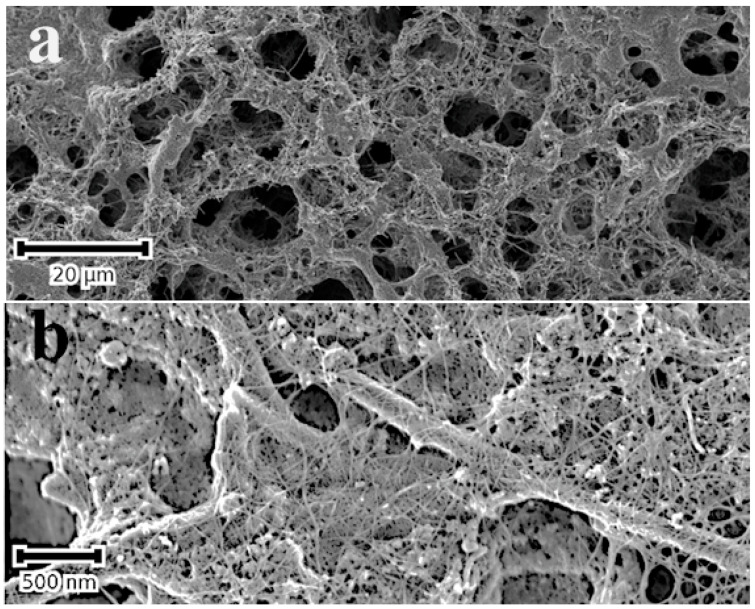

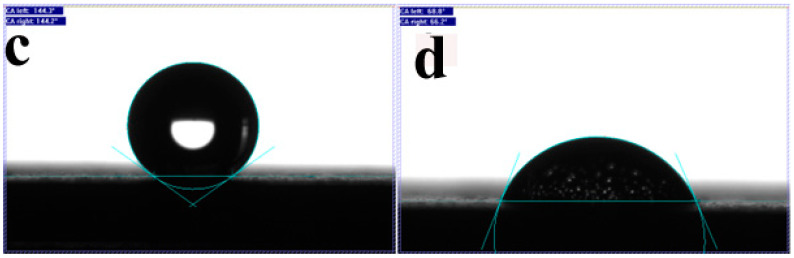
SE micrographs of a porous C-MWCNTs composite on a stainless- steel substrate before (**a**) and after modification with a layer of SWCNTs (**b**). Notice the fine web of SWCNT bundles on the surface in (**b**); (**c**,**d**) are the water contact angles (using the sessile drop method) of the films before (**c**) and after modification with SWCNTs (**d**); WCAs are 144° and 68°, respectively.

This method of surface modification is, in principle, very simple and easily applied to suitable substrates that can withstand temperatures of up to 300 °C. The substrate is heated to a temperature slightly above 290 °C (the Leidenfrost temperature) and directly quenched into the aqueous suspension/precursor solution of the targeted material with which the surface is to be modified. This applies equally to a colloidal solution of SWCNTs and aqueous solutions of noble metal ions. After quenching the substrate in the colloidal solution of SWCNTs, the porous nanocarbon surface exhibits a closely knit web of SWCNT bundles, shown in Figure 4b. As will be discussed below, the SWCNT film changes the wetting behaviour of the nanocarbon surface, shown in Figure 4c,d, and, consequently, the stability of Leidenfrost layer. Additionally, the SWCNTs tend to template the nucleation and growth of the NMNPs imparting a better dispersion to them on the surface.

**Figure 5:**
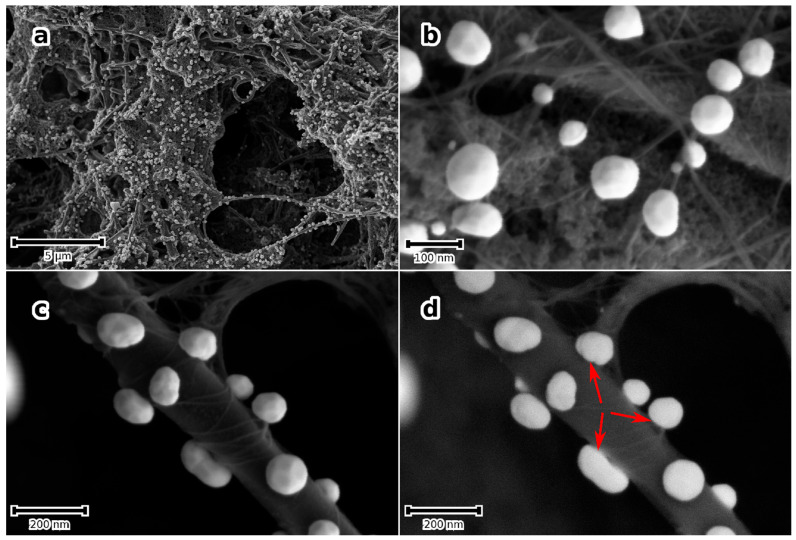
(**a**–**c**) SE micrographs of porous nanocarbon-MWCNT-composite films that modified first with SWCNT and next with Au-NPs. (**a**) Overview, (**b**,**c**) higher magnification micrographs at different locations showing Au-NPs @ SWCNT bundles that appear to wrap one MWCNT in (**c**). (**d**) ESB micrograph of (**c**) depicting a “continuous” interface between the SWCNT and the NPs (arrows).

When the SWCNT modified porous nanocarbon film is quenched from 300 ± 10 °C into an aqueous solution of 2 mM of HAuCl_4_, a high density of Au-NPs that randomly distribute on the hierarchical surface is obtained, shown in Figure 5a. A distribution of particle size, from a few tens of nanometre to 200 nm, may be noticed, and most of the particles decorate the SWCNT bundles, shown in Figure 5b. Further, the high-magnification micrographs, shown in Figure 5c,d, show a rather continuous interface between the Au-NPs and the underlying nanostructures, suggesting a good adhesion of the NPs.

**Figure 6:**
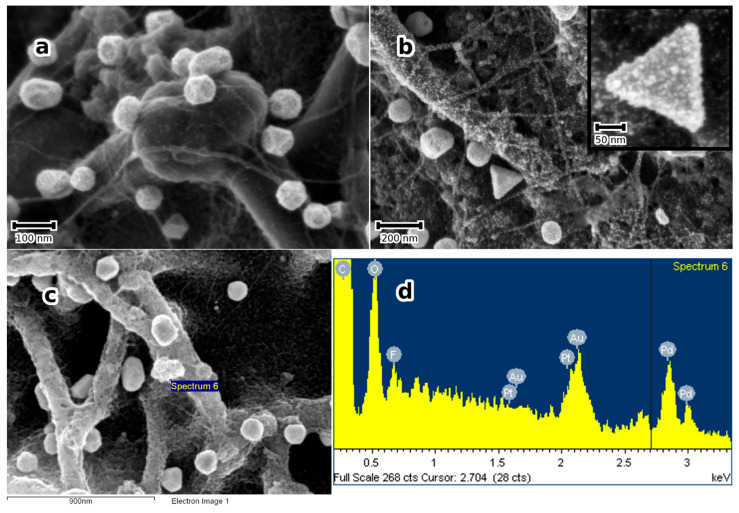

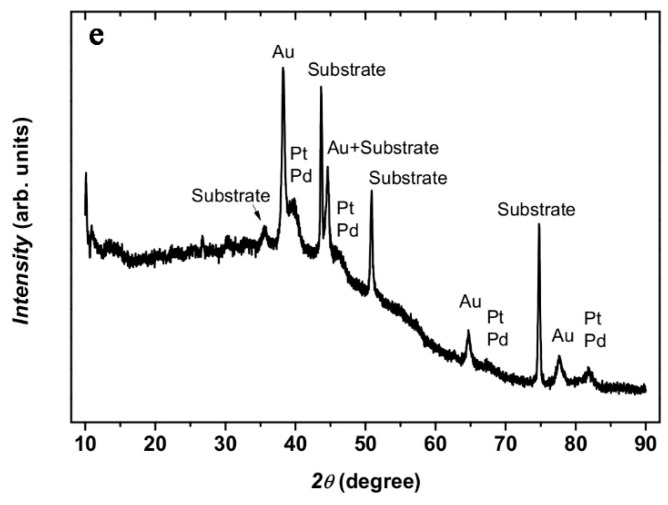
(**a**) SE micrograph of a substrate treated as in Figure 1, but sequentially modified with Au and Pt-NPs. (**b**) SE micrograph of a sample sequentially modified with Au-, Pt- and Pd-NPs (The inset shows a multi-material NP at higher magnification). Notice the NPs decorating the SWCNT bundles; (**c**,**d**) show an SE micrograph and the corresponding EDS spectrum of the Au-, Pt- and Pd-trimetallic NPs demonstrating the presence of the three noble metals, additionally to the substrate’s elements; (**e**) corresponding XRD patterns in grazing incidence mode.

Subsequent treatment of the Au-NPs containing substrates in the same manner using an aqueous solution of H_2_PtCl_6_ yields bimetallic Au-Pt-NPs, where Pt partly reduces on Au-NPs leading to a rough morphology of the NPs, shown in Figure 6, and partly forms Pt-NPs on the substrate surface, decorating SWCNTs.

Bimetallic Au-Pt, Au-Pd, and Pt-Pd as well as Trimetallic Au-Pd-Pt NPs are obtained in the same manner by sequentially treating the substrate in aqueous solutions of the noble metal ions. This is clearly shown in Figure 6b and is confirmed by EDS analyses in Figure 6c,d. At the same time, the XRD patterns, shown in Figure 6e, show two distinct peaks between 38 and 42° (2θ), where the 38°peak can be attributed to Au (or a solid solution of Au with small amounts of Pt and/or Pd, because the Au peak shift is marginal) and the other rather to a solid solution of Pt-Pd, because only one broad peak could be observed. Estimation of the Pt particle size in Au-Pt and Pt-Pd in Au-Pt-Pd using the Scherrer Formula and Lorentzian peak deconvolution of the 111 peak) yields approximately 5 nm, which is in good agreement with the SEM estimations. The chemical composition of the Pt-Pd-NPs can be determined from the peak shift, assuming continuous solid solution and Vegard’s law. Using the 311 peak, a concentration of Pd of approximately 30 at. % was obtained. From these results, it may be concluded that the trimetallic NPs effectively consist of Au supporting a Pt-Pd alloy NPs. This result appeals to some comments: the formation of Pt-Pd-NPs directly from solution at such low temperatures as in the present work may be explained by the exothermic heat of mixing pertaining to the Pt-Pd-system, where ordering is expected [33]. Further, the sub-10 nm size of the particles means a very short mean diffusion length for chemical diffusion. In the whole, it is suggested that the formation of Pt-Pd-alloy NPs is favoured both thermodynamically and kinetically.

**Figure 7:**
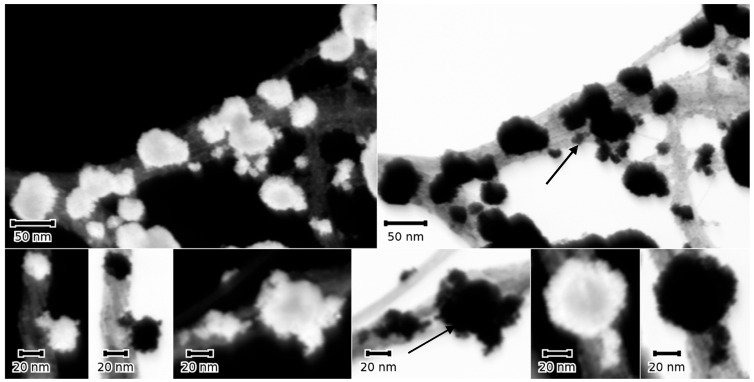
A selection of dark (DF) and bright field (BF) STEM micrographs of a sample sequentially modified with SWCNT, then Au-, Pt-, and Pd-NPs. The micrographs show bigger Au-NPs with tails of smaller Pt and Pd (arrows). The particles adhere to SWCNT bundles that are clearly visible in the BF micrograph, top panel, right image. The DF micrographs show a slight contrast difference between the large and small NP tails.

To further confirm the multi-particle character of the NMNPs, SWCNTs were modified with trimetallic NPs following the procedure described in the experimental section. The selection of particles in the STEM micrographs, seen in Figure 7, suggest bigger particles, probably Au-NPs; the smaller ones are attached to these particles as discrete NPs (see arrows in Figure 7).

## 4. Discussion

Although the mechanisms governing the formation of NPs are similar in both processes, the mechanisms related to sonochemically mediated NPs formation are discussed first here.

Reduction of metal ions in aqueous solutions via ultrasonic radiation have been discussed in detail, and the discussions mainly involve the generation of highly reducing radicals [34]. With respect to the information about noble metal NP formation that has been reported so far, the presence of organic molecules or surfactants was found to be indispensable for the generation of NPs in solution [30,33]. The direct reduction of NPs on a substrate via sonochemistry has been, however, only marginally addressed. In most cases, sonochemistry has been appended to electrochemistry to control the formation of NP catalysts [35]. In the present paper, it is shown that no NPs form when the aqueous solution containing solely ${\mathrm{AuCl}}_{4}^{-}$ complex is sonicated, which agrees with what was published to date [30,34]. Therefore, the generation of reducing radicals from additives to the aqueous solution is a prerequisite to the reduction of the metal ions in a solution. Of more practical interest, however, is the direct reduction of the particles on a substrate surface which should yield substrate-supported catalysts. In the absence of any additives, as is the case in this work, the reducing radicals must have been generated at the interface between collapsing cavities and the substrate surface. This must be the condition sin e qua non for the metal NPs to be reduced on surface sites. In previous work on the effects of ultrasonic radiation on graphite, Guittonneau et al. [36] had shown that high power ultrasonic radiation may decompose graphite, yielding different organic products down to phenols, acids, and CO_2_ in the last stadium. Based on their work, a nanocarbon substrate should therefore generate reducing radicals at its surface when subjected to ultrasonic radiation. This was the main impetus for the present work, which indeed demonstrates that, in the presence of a nanocarbon substrate noble metal ions are reduced on the surface to yield supported NMNPs. This, in turn, indirectly points to the formation of reducing radicals, despite the relatively moderate ultrasonic power and the short sonication time. It is surmised that sonication imparts enough power to the cavitation bubbles to create hot spots at the nanocarbon surface where organic radicals could be generated; the noble metal ions are thought to be reduced at those hot spots, followed by nucleation and growth, as depicted in Equations (1)–(4) [30,34].

H_2_O → ^•^OH + ^•^H (1)

^•^OH + RH → ^•^R (^••^R) + H_2_O (2)

3(^•^R,^••^R) + Au^3+^ → Au^0^ (3)

nAu^0^ → Au_n_^0^ (4)

where (^•^R,^••^R) are reducing radicals at the nanocarbon surface. ^•^OH is from water sonolysis. Au- reduction follows Equation (3) and cluster formation Equation (4).

Another substrate that is shown to be propitious to NP surface attachment is TiN. From Equation (1), the radicals generated by the sonolysis of water may lead to Equation (5)

TiN + ^•^OH → ^•^NH2 (^••^NH) + Ti-O_x_ (5)

3^•^NH_2_ + Au^3+^ → Au^0^ (6)

Metal cluster formation follows Equation (4).

Considering the high temperatures and pressures inside the collapsing cavitation bubble, the highly reactive ^•^OH (and ^•^H) radicals can generate ^•^NH2 (^••^NH) reducing radicals, leading to the reduction of the metal ions together with the oxidation of the TiN surface. TiN is indeed easily oxidized at temperatures down to 350 °C [37,38], with the formation of nitrogen.

Following this reasoning, it is expected that other substrates, such as diamond and DLC (diamond-like carbon) coatings, as well as other nitrides and carbides, may also be surface modified with noble metal ions via sonochemistry, opening new opportunities for novel catalysts (more on this subject in the next paragraph).

The mechanisms governing the formation of NMNPs in the second process are similar to those discussed above. As mentioned in the experimental section, the nanocarbon surface is first modified with hydrophilic SWCNTs via quenching the substrate from approximately 300 °C into an aqueous solution of polyethylene glycol functionalized SWCNT (colloidal suspension). This results in a well-adhered network of SWCNT bundles, which changes the wetting behaviour of the nanocarbon from hydrophobic to hydrophilic (see Figure 4). It is surmised that the SWCNTs are transported through the Leidenfrost layer onto the surface where they form an adherent network, as evidenced by the SEM micrograph of Figure 4b. The attachment of SWCNTs to the surface is probably via micro retention because of the rough and porous morphology of the surface, but covalent bonding might also be operating due to the possibility of radical formation.

**Figure 8:**
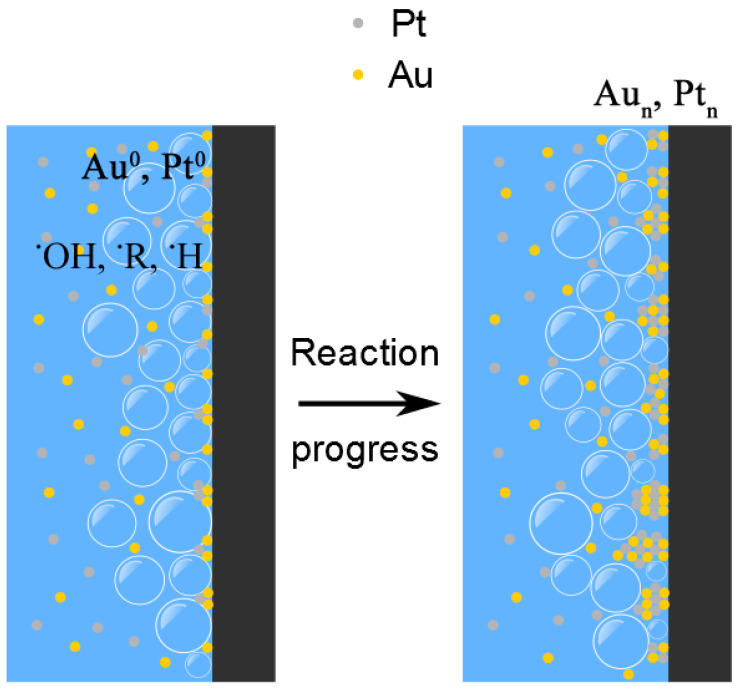
Schematic description of the mechanisms suggested for the formation of NMNPs on the nanocarbon surface. Cavitation bubbles collapsing on the substrate surface (black) lead to the formation of reducing radicals (^•^R, ^••^R, ^•^OH) at the interface. The ensuing reduction of the metal ions occurs at the interfacial sites with the formation of Au^0^, Pt^0^. Nucleation and growth of metal clusters follow as reactions progress.

According to Vakarelski et al. [39], hydrophilic surfaces promote violent bubble nucleation, growth, and collapse when quenched from temperatures above the Leidenfrost temperature. This is exactly what occurs when the SWCNT-modified nanocarbon surface is heated again to 300 °C, then quenched in an aqueous solution of noble metal ions (as described above, the presence of this SWCNT film on the surface changes the WCA from 140° to approximately 67°). This results in the SWCNT bundles becoming decorated with NMNPs (Figure 6b). The mechanisms underlying these processes entail the formation of reducing radicals at the substrate/bubble interface with ensuing transfer of charge to the metal ions; nucleation and growth follow, similarly to what was discussed above for sonochemistry. This is schematically sketched in Figure 8.

**Figure 9:**
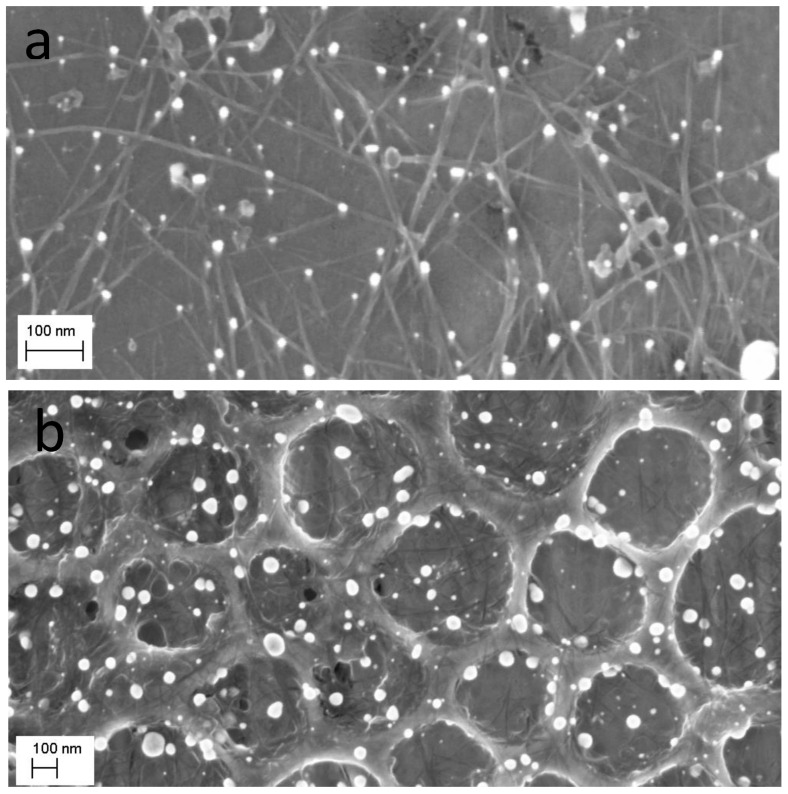
SE micrographs of two TiO_2_ film surfaces modified with SWCNTs and subsequently with Au-NPs. In both micrographs, the SWCNT layer is visible and appears to function as a template for the formation of NPs (**a**). In (**b**), a porous TiO_2_ film was used and the procedure for Au-NPs was repeated twice, which resulted in larger and densely distributed Au-NPs.

The compelling conclusion of the discussion above is that any surface that can be modified with carbon materials, e.g., CNTs, should be eligible for NMNP modification using one of the processes above. This is indeed what has been achieved with TiO_2_ films that were processed using sol-gel on a silicon substrate [40]. These films were first modified with SWCNTs, as indicated above, and subsequently with Au-NPs. As Figure 9 clearly demonstrates, a high density of Au-NPs form on the surface, mainly templated by the SWCNT bundles. Such nanocomposites combine three fundamentally important functional materials: CNTs that are known for their outstanding electronic properties [41]; Au-NPs that are mostly known for their unique plasmonic absorption [42]; and TiO_2_, a wide band-gap semiconductor oxide that can be excited with near UV radiation (ca. 320 nm) to generate electron-hole pairs [43]. The combination of these functionalities is expected to endow the nanocomposite with better properties for several applications including, among others, plasmon-enhanced photocatalysis for visible light photocatalysis [43,44,45] and sensing [46,47]. It has also been shown that TiO2-Au-NPs can act as potent catalysts for hydrogen and acetone formation from propanol, particularly in the presence of a Pt-NP-co-catalyst on TiO_2_ [48], which can be readily achieved with both methods depicted here.

There are good reasons to believe that the nanostructured substrates used in this work are crucial to the density and dispersion of the NMNPs. The spike-like crystallites of the TiN surface, shown in Figure 3a, and the highly porous nanocarbon-MWCNTs, Figure 4a, are likely prone to cavitation-driven oxidative processes and generation of reducing radicals because of their extremely high surface area and the well-known reactivity of such surfaces. This also applies for surfaces that are nanostructured with SWCNTs, as evidenced in the case of TiO_2_ films. The implications of this work are wide ranging. As mentioned above, an important class of functional materials, such as nanocarbon-based materials, carbides, and nitrides, are expected to be easily surface-modified with NMNPs in straightforward and rapid processes. New functionalities may be exploited from the coupling of the specific properties of NMNPs (electronic, catalytic, plasmonic, etc.) to those of the supporting substrates, e.g., [49].

As mentioned earlier, the main advantages of the methods described here over other methods lie in the easiness and rapidity of processing (the processing time may be as short as 60 s) of a high density of NPs on the selected substrates. However, some aspects may be improved, such as the control of particle shape and crystallography, as this is crucial to catalytic applications, e.g., [50], and nanoalloy composition. Controlling particle shape and crystallography may be achieved via an appropriate choice of surfactant chemistry and concentration (see reference [50]) but this will involve more chemicals and processes to avoid catalyst site poisoning by chemical residues. Control of nanoalloy composition may be achieved via a proper understanding of the processes governing atomic transfer from solution to surface, depending on solution chemistry and process parameters. In both cases, work is underway and will be the subject of a future publication.

## 5. Application to Electrocatalysis

One of the obvious applications of the NMNP multi-materials supported on noncarbon is in electrocatalysis. Preliminary experiments were conducted for methanol and formic acid electro-oxidation using Au-Pt and Au-Pd-Pt-NPs.

**Figure 10:**
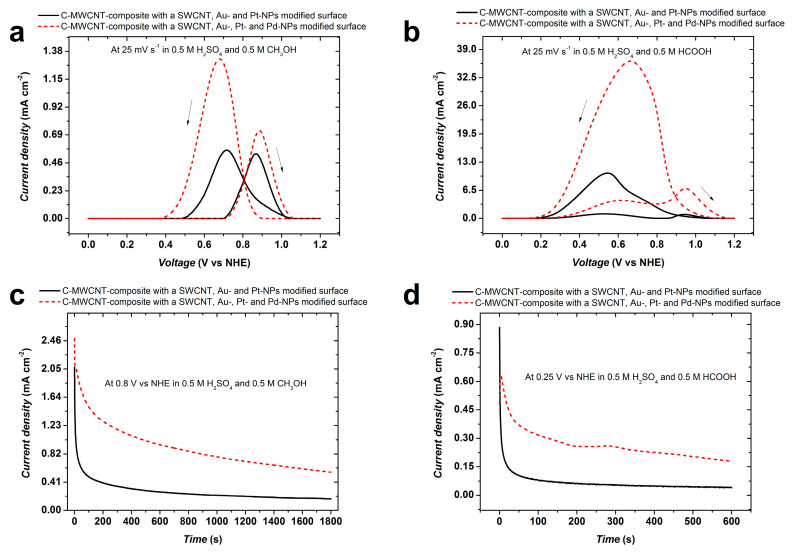
Current density (current normalized by the geometrical surface area) vs. voltage of two sample electrodes in acidic solutions of methanol (**a**) and formic acid (**b**). The full line curves correspond to the Au-Pt, the dashed line curves to the Au-Pt-Pd-NPs modified electrodes; (**c**,**d**) are Chronoamperometric measurements in methanol and formic acid solutions, respectively.

Figure 10 compares the electrocatalytic oxidation activity of the multi-material nanostructures in acidic solutions of methanol and formic acid. For both electrolytes, a substantial improvement of the electrocatalytic activity is obtained with the trimetallic NPs. However, the reverse oxidation peaks are larger than the forward peaks, indicating that, under acidic conditions, oxidation proceeds following the indirect path with the formation of intermediate adsorbates [51]. For methanol oxidation the peak current density (related to the geometric surface area) amounts to 1.32 mA cm^−2^. A rough estimate of the mass of Pt using image analysis and considering the mean particle size and amount of Pt in the PtPd-NPs (from the XRD data) yields approximately 2 µg cm^−2^. Using this value, the peak current density normalized by mass of Pt amounts to approximately 660 A g_Pt_^−1^. Formic acid oxidation shown in Figure 10b exhibits a similar behaviour but at rather higher peak current densities. For both methanol and formic acid electrooxidation, chronoamperometric measurements also show that the trimetallic NPs perform better than the bimetallic ones (see Figure 10c,d). These outstanding electrocatalytic properties demonstrate that the present catalysts are among the best that have been described in the literature [52,53,54,55], and they could pave the way to a new generation of efficient and cost-effective catalysts with applications beyond those described here, e.g., for ground water remediation [56]. There are good reasons to believe that the particular structure of these catalysts that consists of Pt-Pd-alloy-NPs covering Au-NPs is responsible for their high performance. Considering that the surface energy of Pd is lower than that of Pt, Pd-surface segregation may be expected, probably one monolayer [57], due to the low temperature involved in the process. As has been reported before, and based on density functional theory calculations, this is a favourable structure for boosting catalyst performance, probably as result of the so-called ligand effect [58].

## 6. Conclusions

Two powerful, environmentally sustainable and material efficient methods for the synthesis of self-organized noble metal nanoparticles (NMNPs) and multi-materials thereof are presented. The methods rely on the reduction of metal ions directly on nanocarbon and TiN substrate surfaces entailing the formation of supported NPs. First, the synthesis of highly dense NMNPs using a simple laboratory ultrasonic device is presented. It is shown that supported Au-NPs directly form on nanocarbon and TiN surfaces solely by sonicating the substrates in a 2 mM HAuCl_4_ solution for a short time without addition of other chemicals. In the second method, a nanocarbon surface is heated to a temperature slightly above the Leidenfrost temperature of 290 °C and directly quenched in the aqueous solutions of 2 mM HAuCl_4_. The formation of adherent, highly dense Au-NPs is also demonstrated. Sequential heating and quenching in separate solutions of ions of Pt and Pd result in the formation of bimetallic and trimetallic NPs. Because no chemical additives were added to the solution to catalyse NP formation, the results are discussed in terms of reducing radicals (^•^R; ^••^R; on nanocarbon and ^••^NH; ^•^NH_2_ on TiN) generation at the interface between substrate and impinging bubbles. It is surmised that enough energy is stored in the bubbles to entail reducing radical formation. The sites of the collapsing bubbles determine the self-organization of the NPs on the surface. Consequently, it is exemplarily demonstrated that, using TiO_2_ films, any surface that could be modified with a nanocarbon layer is eligible for noble metal ion reduction and NMNP formation.

Finally, the bimetallic and trimetallic NPs on nanocarbon were tested for their electrocatalytic activities in acidic solutions of methanol and formic acid in preliminary experiments. It was shown that these materials belong to the best catalysts reported in the literature.

## Data Availability

Data are unavailable due to privacy restrictions.
